# United States Normative Attitudes for Pursuing Parenthood as a Function of Gender, Sexual Orientation, and Age

**DOI:** 10.3389/fpsyg.2021.772252

**Published:** 2022-01-05

**Authors:** Doyle P. Tate

**Affiliations:** Department of Psychology, Penn State Scranton, Dunmore, PA, United States

**Keywords:** lesbian, gay, parenthood, norms, attitudes

## Abstract

Decisions about whether or not to become a parent are significant parts of normative human development. Many studies have shown that married different-sex couples are expected to become parents, and that many social pressures enforce this norm. For same-sex couples, however, much less is known about social norms surrounding parenthood within marriage. This study examined injunctive norms and descriptive norms for the pursuit of parenthood as a function of age, gender, and sexual orientation. Participants in an internet survey included 1020 (522 heterosexual, 498 lesbian/gay) cisgender people from across the United States Findings showed that norms, especially descriptive norms, for the pursuit of parenthood for heterosexual people were much stronger than those for lesbian women and gay men, and that norms for lesbian women were stronger than those for gay men. These differences were more pronounced for older, heterosexual, and male participants. However, lesbian and gay participants, especially gay men, reported that lesbian and gay people ought to become parents to the same extent as heterosexual people. Overall, the results indicated that, regardless of sexual orientation, adults report that lesbian and gay married people ought to become parents, but that they expect only a minority of these couples will pursue parenthood. This research provided a glimpse into how Americans are envisioning family formation among same-sex couples today.

## Introduction

Decisions about whether to become a parent are important parts of normative human development (Hoffman, 1975; Langdridge et al., 2005; Jaffe and Diamond, 2011). Married different-sex couples are expected to become parents, and many social pressures serve to enforce this process (Jamison et al., 1979; Mueller and Yoder, 1997, 1999; Kopper and Smith, 2001; DeJean et al., 2012; Ashburn-Nardo, 2017). For same-sex married couples, however, less is known about the social processes and norms involved in the pursuit of parenthood. However, there have been well-documented disparities within parenting intentions, desires, and achievement for lesbian and gay individuals compared to heterosexual individuals (Gates et al., 2007; Patterson and Riskind, 2010; Riskind and Patterson, 2010; Riskind and Tornello, 2017; Tate et al., 2019; Tate and Patterson, 2019). More and more lesbian and gay people are choosing to parent within same-sex relationships after coming out, and this trend is expected to increase with changing social climates (Rabun and Oswald, 2009; Riskind and Patterson, 2010; Goldberg et al., 2012; Bauermeister, 2014). This study investigated norms for parenthood as a function of age, gender, and sexual orientation.

Much of what is known about fertility and decision-making processes regarding fertility has been based on studies of heterosexual people (Ashburn-Nardo, 2017; Gato et al., 2017). Most adolescents and young adults have reported that achieving parenthood is important to them (Riskind and Patterson, 2010; Ashton-James et al., 2013), and most desire parenthood (Hagewen and Morgan, 2005; Ashton-James et al., 2013; Riskind and Tornello, 2017). A significant normative assumption for the framework of this study was that adults want to become parents in the context of a long-lasting romantic partnership or marriage (Langdridge et al., 2005; Ashburn-Nardo, 2017; Gato et al., 2017; Tate et al., 2019). However, many people do become parents while single or otherwise outside of marriage. The value of achieving parenthood for most of the population makes the mechanisms of voluntary fertility decision-making important to understand.

It has become clear that lesbian and gay individuals intend to become parents less often than do their heterosexual peers (Patterson and Riskind, 2010; Riskind and Tornello, 2017; Tate et al., 2019). Working with a nationally representative sample, Riskind and Patterson (2010) studied data from childless adults and reported 54% of gay men compared to 75% of heterosexual men stated a desire to achieve fatherhood, while 37% of lesbian women compared to 68% of heterosexual women stated a desire to achieve motherhood. In other words, lesbian and gay individuals were less likely than their heterosexual peers to express a desire or intention to become parents. Gay and lesbian individuals are also less likely to expect to achieve parenthood compared to heterosexual individuals (Tate and Patterson, 2019). In both United States and Israeli samples, gay and lesbian individuals tend to report desiring parenthood more than they expect to achieve it (Shenkman, 2012; Tate and Patterson, 2019). Why do lesbian and gay adults report lower aspirations for parenthood? Many studies have investigated possible reasons as to why differences exist (Allen and Demo, 1995; Brown et al., 2009; Rabun and Oswald, 2009; Riskind et al., 2013; Gato et al., 2017; Tate et al., 2019), but none have examined differences in the injunctive and perceived descriptive norms surrounding parenthood. Differences in parenthood aspirations as a function of sexual orientation may stem from differences in norms surrounding parenthood for lesbian and gay individuals in comparison to heterosexual individuals.

Normative social influence encourages individuals to conform to norms and the behavior of others to be liked and accepted (Asch, 1956; Cialdini et al., 1991; Rimal and Real, 2003). Two significant aspects of normative influence are perceived descriptive norms and injunctive norms. Perceived descriptive norms are the degree to which people think that others perform or partake in a behavior (Cialdini et al., 1991; Rimal and Real, 2003), whereas injunctive norms are how much individuals think that others *should* perform or partake in a behavior (Cialdini et al., 1991; Rimal and Real, 2003). This could be connected to family formation in that many social normative pressures act on heterosexual individuals regarding family formation (Jamison et al., 1979; Mueller and Yoder, 1997, 1999; Kopper and Smith, 2001; DeJean et al., 2012; Ashburn-Nardo, 2017). In other words, people tend to believe parenthood will be and should be achieved by “normal” married different-sex couples.

The normative social aspects of family formation have been found to begin early in life within reproductive stories starting in childhood (Jaffe and Diamond, 2011). During this time, children develop the ideals which will structure their life course decisions and their perceptions of life events as being either “on-time” or “off-time” (Mueller and Yoder, 1997, 1999; Jaffe and Diamond, 2011). For many, the sequence described in the nursery rhyme “first comes love, then comes marriage, then comes baby in a baby carriage” became ingrained early in life (Mueller and Yoder, 1997, 1999; Jaffe and Diamond, 2011). In fact, breaking from this sequence could have negative effects. For example, single parents are judged harshly compared to married parents (Mueller and Yoder, 1997, 1999; Kopper and Smith, 2001; DeJean et al., 2012). Thus, following the social script for how to achieve parenthood could be seen as a process starting early in life.

These social normative processes continue into adulthood. For instance, married heterosexual men and women are viewed negatively when they choose not to become parents, and they may face societal backlash (Jamison et al., 1979; Calhoun and Selby, 1980; Ashburn-Nardo, 2017). For example, Jamison et al. (1979) studied perceptions of women and men who are voluntarily childless. They found that voluntarily childless women were viewed as less sensitive, less loving, and less typical of an American woman than those who were mothers. In addition, these childless women were rated as less happy, less well-adjusted, and less likely to be content at age 65. Voluntarily childless men were also viewed negatively, being regarded as more selfish and less typical, less well-adjusted, less sensitive, less loving, and less fulfilled than were fathers (Jamison et al., 1979). More recent studies have reported similar findings (Ashburn-Nardo, 2017). For instance, Ashburn-Nardo (2017) found that the couples who decided not to become parents elicited more reported moral outrage than those who intended to become parents. Greater moral outrage toward voluntary childlessness partially explained why participants reported that married couples who had chosen not to become parents would be less fulfilled in life. Thus, the existence of norms for parenthood in adulthood has a profound impact on the attitudes held about others.

Lesbian and gay individuals are less likely than heterosexual individuals to intend to become parents (Patterson and Riskind, 2010; Riskind and Tornello, 2017; Tate et al., 2019). Yet, little is known about how normative influence is involved in the parenthood decisions of lesbian and gay individuals. Social contexts are important in lesbian and gay family formation. Contextual factors, such as having a partner who wants children, and social support from family, friends, and LGBT networks, have been associated with higher parenthood intentions among lesbian and gay individuals (Berkowitz and Marsiglio, 2007; DeMino et al., 2007; Gianino, 2008; Goldberg, 2010; Goldberg et al., 2012). Conversely, negative contextual factors, such as legal, medical, and social barriers to parenthood and anticipated stigma surrounding parenthood, have been associated with a lower likelihood to parent for lesbian and gay individuals (Allen and Demo, 1995; Brown et al., 2009; Rabun and Oswald, 2009; Riskind et al., 2013; Gato et al., 2020; Shenkman, 2021). Thus, many contextual factors may influence parenthood intentions among lesbian and gay individuals.

In addition, contextual factors have been found to influence parenthood intention in similar ways regardless of gender or sexual orientation. A recent study, using a large national dataset from the United States, found that all the demographic, personal, and social contextual variables examined that were associated with parenthood intentions of heterosexual adults were also similarly associated with parenthood intentions of lesbian and gay adults (Tate et al., 2019). Having more close friends, closer relationships with parents, and greater reported relationship permanence was associated with a greater likelihood of parenthood intention regardless of sexual orientation. Differences in these factors also explained part of the disparity in parenthood intention for lesbian and gay adults and their heterosexual counterparts, especially for lesbian women. Lower reported relationship permanence among gay men and lower parental closeness among lesbian and gay individuals compared to their heterosexual counterparts explained part of the disparity in parenthood intention as a function of sexual orientation (Tate et al., 2019). Little is known, however, about the social norms for parenthood among lesbian and gay individuals.

Historically, lesbian and gay individuals were not able to undertake legal marriage or become parents within a same-sex context. However, a movement for LGBT rights has made this possible for sexual minority individuals within the United States (D’Augelli et al., 2007; Tankard and Paluck, 2017). In fact, it has been found that many contemporary lesbian and gay youth envision a seemingly “normative” future that involves getting married and becoming parents within a same-sex relationship (D’Augelli et al., 2007). Because of recent changes in marriage equality and family laws, younger cohorts of lesbian and gay individuals may be experiencing more favorable environments for parenthood than those experienced by older cohorts (Riskind and Patterson, 2010; Baiocco and Laghi, 2013; Tornello and Patterson, 2015; Costa and Bidell, 2016). Yet, societal expectations about whether married same-sex couples ought to pursue parenthood have not been studied.

Norms and normative social influence may change over time. For instance, interracial marriage has been legalized throughout the United States only since 1967. Since then, interracial marriage has become more socially acceptable, and this trend is expected to increase over time (Garcia et al., 2015). Similarly, there have been recent legal changes for same-sex marriage and parenthood that may have an impact on normative influence and behavior.

Norms about same-sex marriage changed after marriage equality legislation (Tankard and Paluck, 2017), and many sexual minority youths have been found to envision marriage and parenthood within a same-sex context (D’Augelli et al., 2007). It would also be important to consider differences in historical and generational contexts that may be influencing how people of different ages perceive parenthood among same-sex couples. For instance, it has only been since 2003 that sexual activity between consenting same-sex partners of legal age has been legal nationwide (Lawrence v. Texas, 2003), and same-sex marriage has only been legal nationwide since 2015 (Obergefell v. Hodges, 2015). Not only laws but also attitudes toward lesbian and gay people throughout the United States have rapidly become more favorable (Avery et al., 2007; Pew Research Center, 2017). Public opinion toward same-sex couples has become progressively more positive since 1977, and this trend is expected to continue (Avery et al., 2007). Because of differences in historical and generational contexts, it is important to examine normative social influence among people of different ages.

There has also been data suggesting that views about lesbian and gay parenthood may be more positive among younger than among older Americans. For instance, in a poll conducted by the Pew Research Center (2012), 67% of Americans between the ages of 18 and 29 thought that lesbian and gay people should be allowed to (legally) adopt children. This percentage decreased as the age of the respondents increased; 56% of those between the ages of 30 and 49, 47% of those from 50 to 64 years old, and only 35% of those who were older than 65 thought that lesbian and gay individuals should be allowed to adopt children (Pew Research Center, 2012). These findings helped support the idea that lesbian and gay parenthood may be more acceptable among younger Americans, but they did not indicate the current state of injunctive or descriptive norms for lesbian and gay parenthood.

Moreover, it is important to consider that social norms and expectations around parenthood are changing for everyone. For instance, birthrates have been decreasing throughout much of the developed world, with more and more people choosing to be childfree (Tanaka and Johnson, 2016). With the advent of more effective birth control methods, parenthood has become more of a choice and an optional pathway for a fulfilling life for heterosexual people (Peterson, 2015). However, choosing not to pursue parenthood in cultural settings where parenthood is socially expected and encouraged could be detrimental to well-being and life outcomes (Tanaka and Johnson, 2016). Moreover, many younger people within the United States lack economic resources and even have overwhelming debts that prevent the pursuit of parenthood, and this issue has loomed larger for contemporary younger adults than in past generations (Nau et al., 2015). Thus, younger people in the United States may view parenthood as something that *some* people should do, but also acknowledge that not every couple can become parents.

This study examined norms for parenthood among lesbian and gay married couples across multiple age groups. The existence of a norm for parenthood is still an unknown for same-sex married couples. This study aimed to assess the perceived descriptive and injunctive norms for lesbian, gay, and heterosexual parenthood as a function of participants’ sexual orientation, gender, and age. There were three main research questions: (1) How do norms for parenthood differ depending on the type of couple pursuing parenthood? (2) How do these differences depend on the sexual orientation, gender, and age of those responding to the norm measures? (3) What do these results indicate about the norms for the pursuit of parenthood among married same-sex couples?

For the first question, it was expected that norms for heterosexual parenthood would be stronger than those for lesbian and gay parenthood and that norms for lesbian parenthood would be stronger than those for gay parenthood among all participants. This hypothesis is based on the fact that lesbian and gay parenthood is usually viewed less favorably compared to heterosexual parenthood (McCutcheon and Morrison, 2015; Gato et al., 2017), but that lesbian parenthood has been rated more favorably than gay parenthood (McCutcheon and Morrison, 2015). For the second research question, it was expected that lesbian and gay individuals would endorse stronger norms for lesbian and gay parenthood than would heterosexual individuals. It was also expected that women would rate stronger norms for lesbian and gay parenthood than would men, and that younger participants would endorse stronger norms for lesbian and gay parenthood than would older participants. These hypotheses were based on findings from the Pew Research Center (2012) suggesting that women and younger people are more in favor of lesbian and gay parenthood than are men and older people. Finally, for the third research question, it was expected that participants, especially those who are younger, would rate parenthood as something that married lesbian and gay couples should do, but also something that not that many lesbian and gay couples would actually do.

## Materials and Methods

### Participants

Participants included 1020 lesbian, gay, and heterosexual individuals within the United States who were above the age of 18 (267 lesbian women, 231 gay men, 260 heterosexual women, 262 heterosexual men). Both childless individuals and those who were parents were eligible for inclusion. Participants identified as cisgender male or female. Age was categorized into four groups defined as Early Adults = 18–24 years, Young Adults = 25–34 years, Younger Middle Adults = 35–44 years, Older adults = 45 + years (Costa and Bidell, 2016). Middle adulthood was split into two groups because of the biological and social factors surrounding parenthood that differ within this period (Costa and Bidell, 2016).

*A priori* power analyses assuming 80% power were conducted for 2 mixed analyses of variances (ANOVAs) with R statistical software to calculate sample sizes. Four hundred and four participants were needed to detect interaction effects between within- and between-subjects’ factors for the 2 × 2 × 4 ANOVA with three repeated measures. There were 16 groups, and 404 divided by 16 is 25.25. Thus, 26 people were needed for each group. This translated into 104 people per age group (26 gay men, 26 lesbian women, 26 heterosexual men, and 26 heterosexual women). Thus, the aim was to collect data from at least 416 participants.

Participants included 240 Early Adults (63 lesbian women, 53 gay men, 65 heterosexual women, 59 heterosexual men), 281 Young Adults (75 lesbian women, 71 gay men, 66 heterosexual women, 69 heterosexual men), 256 Younger Middle Adults (77 lesbian women, 50 gay men, 64 heterosexual women, 65 heterosexual men), and 243 Older Adults (52 lesbian women, 57 gay men, 65 heterosexual women, 69 heterosexual men). In all, more than the minimum number needed per group was achieved, and the analyses had over 99% power to detect medium size effects.

Participants were recruited online in the Summer of 2019 using TurkPrime, now called CloudResearch. This system gathered individuals from participant panels made up of heterosexual, gay, and lesbian individuals living across the United States to recruit the demographics needed for the study (Litman et al., 2017). Age quotas were also utilized in date collection to achieve relatively equal samples of participants within the age categories. This research was originally approved by the (masked) Institutional Review board.

Despite the sample being large and diverse, it was not representative of the United States population (see Table 1). Compared to the United States population, a greater proportion of heterosexual participants and lesbian/gay participants were white, *z* = 4.20, *p* < 0.001, *z* = 2.73, *p* = 0.006, respectively. A greater proportion of lesbian and gay participants had a bachelor’s degree or above compared to the United States population, *z* = 8.61, *p* < 0.001. Heterosexual participants did not significantly differ from the overall population of the United States in education, *z* = 0.98, *p* = 0.327.

**TABLE 1:** Sample statistics compared to United States population statistics.

Demographic variable	United States population statistic	Heterosexual participants (*n* = 522, 51%)	Lesbian/gay participants (*n* = 498, 49%)	Population comparisons	Within-sample comparisons
**Race**					
% White alone (not Hispanic or Latino)	60%^1^	69%	66%	P < H*** P < LG**	H ≈ LG
**Gender**					
% Female	51%^1^	50%	54%	P ≈ H P ≈ LG	H ≈ LG
**Education**					
% Bachelor’s Degree or Above	32%^1^	34%	50%	P ≈ H P < LG***	H < LG***
**Family**					
Is a parent	61%^2^	49%	29%	P > H*** P > LG***	H > LG***
Is married	50%^3^	39%	24%	P > H*** P > LG***	H > LG***
**Age**					
18–25	9%^4^	27%	27%	P < H*** P < LG***	H ≈ LG
26–34	12%^4^	23%	26%	P < H*** P < LG***	H ≈ LG
35–54	26%^4^	36%	35%	P < H*** P < LG***	H ≈ LG
55+	29%^4^	14%	12%	P > H*** P > LG***	H ≈ LG

*P represents United States population, H represents Heterosexual Participants, and LG represents Lesbian/Gay participants.*

*^1^U.S. Census Bureau QuickFacts: United States (2019).*

*^2^Pew Research Center for the People and the Press Poll (2013).*

*^3^Geiger and Livingston (2019).*

^4^Population Distribution by Age (2019). **p < 0.01; ***p < 0.001.

Due to the effort to control sample sizes across age categories, heterosexual and lesbian/gay participants were much younger than the United States population (see Table 1). Compared to the United States population, more heterosexual participants were ages 18–25, 26–36, and 35–54, *z* = 14.37, *p* < 0.001, *z* = 7.73, *p* < 0.001, *z* = 5.21, *p* < 0.001, respectively. Likewise, a greater proportion of lesbian and gay participants were ages 18–25, 26–36, and 35–54 compared to the United States population, *z* = 14.04, *p* < 0.001, *z* = 9.61, *p* < 0.001, *z* = 4.58, *p* < 0.001, respectively. Moreover, fewer heterosexual participants and lesbian/gay participants were older than 55 compared to the United States population, *z* = 7.55, *p* < 0.001, *z* = 8.36, *p* < 0.001. With these age differences in mind, a greater proportion of the United States population was married than were lesbian/gay and heterosexual participants, *z* = 11.60, *p* < 0.001, *z* = 5.03, *p* < 0.001. Moreover, a greater proportion of the United States population reported being a parent than did the lesbian/gay and heterosexual participants in this sample, *z* = 14.64, *p* < 0.001, *z* = 5.62, *p* < 0.001. Overall, the recruited sample was not representative of the United States population.

There were also differences within this sample as a function of sexual orientation (see Table 1). More lesbian and gay participants had a bachelor’s degree or above than did heterosexual participants, χ^2^ = 26.79, *p* < 0.001. Moreover, a greater proportion of heterosexual participants reported being married and/or being a parent than did lesbian and gay participants, χ^2^ = 26.47, *p* < 0.001, χ^2^ = 42.72, *p* < 0.001, respectively. In all, there were many differences within the sample based on participant sexual orientation.

### Measures

#### Demographics

Demographic variables measured in the study included sexual orientation, gender, race/ethnicity, age, education level, marriage status, parenthood status. Sexual orientation was assessed by asking participants, “*Which best describes your sexual orientation?*” with “*Heterosexual(“straight”)*,” “*Lesbian/Gay*,” “*Bisexual*,” “*Pansexual*,” “*Asexual*,” and “*These do not describe me. (Please specify how you identify).”* Only those who select “*Heterosexual(“straight”)*” and “*Lesbian/Gay*” were eligible to participate in this study. Gender identity was assessed using the question, “*What best describes your gender?*” Participants had the option to select, “*Male*,” “*Female*,” “*Genderqueer/Gender non-conforming*,” or “*These do not describe me. (Please specify how you identify)*.” Only those who select “Male” or “Female” were eligible to participate. *Trans* identity, for screening purposes, was assessed by asking the question, “*Some people describe themselves as transgender when they experience a different gender identity from their sex at birth. For example, a person born into a male body, but who feels female or lives as a woman. Do you consider yourself to be transgender??*” Participants had the option to select “*Yes, transgender, male to female*,” “*Yes, transgender, female to male,” “Yes, transgender, gender non-conforming,”* or “*No*.” Those who select yes were not eligible for the study.

Race/Ethnicity was assessed by asking, “*Choose one or more racial and ethnic identities that best describes you.*” Participants had the option to respond with “*White*,” “*Black or African American*,” “*Latino/Latina/Latinx*,” “*American Indian or Alaska Native*,” “*Asian (including East Asian, South Asian, etc.)*,” “*Native Hawaiian or Pacific Islander*,” “*Middle Eastern*,” or “*These do not describe me. (Please specify how you identify).* However, for this study, race was coded by collapsing these responses into three groups: “white,” “multiracial,” and “racial minority.” Age was assessed through the question, “*What is your age in years?*”. Participants were asked to type in their age as a whole number 18 or above. This was recoded into four age categories: Early Adults = 18–24 years, Young Adults = 25–34 years, Younger Middle Adults = 35–44 years, Older adults = 45+ years (Costa and Bidell, 2016). Age was not examined as a continuous variable as the findings from age were not linear. Education level was assessed using the question “*What is the highest level of school you have completed or the highest degree you have received?*”. This had 7 levels ranging from “*Less than a high school degree*” to “*Doctoral or Professional Degree (Ph.D., JD, MD).”* These were then recoded into low (high school education or less), medium (some college or other higher education), and high (bachelor’s degree and above) categories, to create a measure of socioeconomic status (Sumontha et al., 2018; Tate et al., 2019). Marriage and parenthood statuses were evaluated by asking, “*Are you currently married?*,” and “*Are you a parent or step-parent?*”, respectively. For both items, participants could answer “*Yes*” or “*No.*” Parental and marital status did not affect eligibility for the study.

#### Injunctive Norms for Parenthood

Individual injunctive norms for parenthood were assessed. Individual injunctive norms indicate how much participants personally feel that individuals should do something (Cruwys et al., 2015). In this case, the normative behavior is the pursuit of parenthood for each of three childless married couple types (gay, lesbian, and heterosexual). A four-item measure from Cruwys et al. (2015) was adapted to assess these (see Appendix A for full measure). Participants were first given instructions that indicate that the couples asked about in this measure were in their 20 and 30 s and within stable and loving marriages. One example of these items, across each within-group condition, is as follows: “*I think that lesbian couples should become parents*,” “*I think that gay male couples should become parents*,” “*I think that heterosexual (straight) couples should become parents*.” Each of the items was scored from 1 = “*Strongly agree*” to 5 = “*Strongly disagree.*”

A Rasch-based Partial Credit Model from Item Response Theory (IRT) was conducted using the “mirt” package in R statistics software to assess these scales, both overall and by the sexual orientation and gender of the participant (Embretson and Reise, 2000; Chalmers, 2012). When examining the scales for participants overall, the main findings were that the items overfit the model for each condition and values of “disagree” and “strongly disagree” were associated with higher theta levels whereas “strongly agree” were associated with theta levels of around 0 (see Table 2). In essence, the items overfitting the model implied that all items were assessing the same concept in very similar ways and that there was very little predictive error. Moreover, the IRT models suggested that it was more difficult to respond negatively to these scales, regardless of the type of couple being examined. In addition, the threshold for the response of “disagree” was never most likely to occur. This indicated that participants who had any disagreement with the items most often would “strongly disagree.”

**TABLE 2:** Findings from Item Response Theory analyses.

Item	*M* (*SD*)	a	b1	b2	b3	b4	Infit	ZInfit	Outfit	ZOutfit
**Heterosexual couple condition**										
Heterosexual (“Straight”) couples pursuing parenthood is a good idea	1.51 (0.79)	1	1.35	3.05	6.36	6.10	0.44	–13.78	0.41	–14.04
I approve heterosexual (“Straight”) couples becoming parents	1.38 (0.71)	1	2.11	3.77	6.34	6.01	0.53	–9.39	0.37	–11.03
I think that heterosexual (“Straight”) couples should become parents	1.76 (0.95)	1	0.54	1.71	5.46	5.56	0.72	–6.85	0.70	–7.26
I believe that heterosexual (“Straight”) couples becoming parents is a good thing	1.50 (0.78)	1	1.45	3.06	6.57	5.93	0.41	–14.65	0.36	–14.73
**Lesbian couple condition**										
Lesbian couples pursuing parenthood is a good idea	1.99 (1.26)	1	–0.04	1.55	4.38	3.48	0.35	–16.70	0.35	–15.81
I approve of lesbian couples becoming parents	1.87 (1.26)	1	0.61	2.21	4.03	3.63	0.38	–14.09	0.35	–12.94
I think that lesbian couples should become parents	2.15 (1.27)	1	–0.54	0.67	3.98	3.83	0.56	–10.97	0.54	–11.05
I believe that lesbian couples becoming parents is a good thing	1.99 (1.26)	1	–0.10	1.67	4.13	3.67	0.32	–18.15	0.33	–17.13
**Gay couple condition**										
Gay male couples pursuing parenthood is a good idea	2.09 (1.31)	1	–0.18	1.09	3.85	3.63	0.38	–16.18	0.36	–15.06
I approve of gay male couples becoming parents	1.97 (1.33)	1	0.49	1.62	3.68	3.61	0.39	–14.12	0.32	–13.16
I think that gay male couples should become parents	2.25 (1.32)	1	–0.78	0.39	3.67	3.51	0.59	–10.19	0.57	–10.19
I believe that gay male couples becoming parents is a good thing	2.09 (1.32)	1	–0.21	1.26	3.83	3.45	0.33	–17.63	0.32	–16.72

*Items are scaled from 1 = “Strongly agree” to 5 = “Strongly disagree.”*

Differences by participant sexual orientation and gender were also assessed, but these findings were not discussed as these analyses also showed the same pattern of results described above, regardless of sexual orientation, gender, or type of couple. For each couple condition, the items showed high internal reliability (Lesbian condition α = 0.97, Gay condition α = 0.97, Heterosexual condition α = 0.91). The Cronbach’s alphas when splitting the sample by sexual orientation were 0.90 and above for every identity in each condition.

Based on these findings, scores for the four-items within each condition were averaged into a single scale measuring injunctive norms for parenthood that had three within-subject conditions, one for each type of couple. These items were also reverse coded such that scale scores ranged from 1 to 5 with higher numbers indicating stronger injunctive norms.

#### Perceived Descriptive Norms for Parenthood

Perceived descriptive norms for parenthood were assessed using a single item adapted from Cho et al. (2015). First, participants were given instructions that clarified that the couples asked about in this item were in their 20 and 30s. The adapted item was, “*What percentage of (type of married couple) in the United States do you estimate will become parents during their marriage?*” with the couple types being gay, lesbian, and heterosexual couples. Participants were shown these categories instead of same-sex male, same-sex female, and different-sex couples, which tend to be more academic terms and less utilized in daily language, in order to increase clarity and reduce confusion among participants. Respondents could answer from 1 = “0%” to 11 = “100%” with 10% intervals based on previous adaptations of the item (Cho et al., 2015). Higher responses represented stronger descriptive norms.

### Procedure

Participants took part in an anonymous online survey. Participants were told that the goal of this study was to evaluate what people thought as “normal” for couples in the United States today. First, to determine their eligibility, participants answered screening questions about age, gender, transgender status, sexual orientation, and whether they lived in the United States. Those who were not cisgender men or women, who did not live in the United States, and those who were not lesbian, gay, or heterosexual were not allowed to continue forward in the survey.

Participants then completed a block of questions that assess descriptive norms for parenthood. Next, participants completed three blocks of questions assessing injunctive norms for parenthood for the three types of couples (lesbian, gay, and heterosexual). The presentation of the injunctive norm question blocks was randomized. Items within each of the blocks were also randomly presented. The randomization of measures and items was done to reduce possible order-effects.

Finally, demographic and other variables of interests were collected at the end of the survey. Participants were then redirected to receive their reward. In addition, participants were debriefed about the nature of the study.

### Plan of Analyses

Statistical analyses were conducted in SPSS 27. Demographic variables were evaluated as a function of gender and sexual orientation using analyses of variances (ANOVAs), chi-square analyses, and generalized linear models (GLMs) to identify possible covariates to use in further analyses (see Table 3).

**TABLE 3:** Differences in demographic variables as a function of gender and sexual orientation.

	Men		Women						
Variable *n* =	Hetero. 262	Gay 231	Hetero. 260	Lesbian 267	Test statistic_Gender_	Test statistic_Sexual Orientation_	Test statistic_GXS._	Differences	Effect size
Education	2.12 (0.05)	2.32 (0.05)	2.06 (0.05)	2.35 (0.05)	*F* = 0.09	*F* = 27.27***	*F* = 0.80	LG > H***	*g* = 0.32
Parenthood status	0.40 (0.03)	0.15 (0.02)	0.57 (0.03)	0.40 (0.03)	χ^2^ = 61.26***	χ^2^ = 50.60***	χ^2^ = 4.91*	HW > HM*** HW > G*** HW > L*** HM > G*** L > G***	*h* = 0.34 *h* = 0.91 *h* = 0.34 *h* = 0.57 *h* = 0.57
Marriage status	0.36 (0.03)	0.22 (0.03)	0.42 (0.03)	0.25 (0.03)	χ^2^ = 1.87	χ^2^ = 26.92***	χ^2^ = 0.12	H > LG***	*h* = 0.35

Degrees of freedom for F values are (1, 1016). Values for χ^2^ are Wald χ^2^. The differences in the proportions for race and the number of each people in each age groups can be found in-text. *p < 0.05, ***p < 0.001.

Because the design had both within- and between-subject elements, analyses had to take both into account. Two linear mixed effect models (LMMs) using the Satterthwaite method of estimating degrees of freedom that controlled for covariates and repeated measures were conducted in lieu of mixed-effect ANOVAs for these analyses (Gałecki and Burzykowski, 2013). Two 2 (heterosexual, lesbian/gay) × 2 (male, female) × 4 (early adult, young adult, early middle adult, and older adult age groups) × 3 (lesbian, gay, and heterosexual couple within-subject conditions) LMM were conducted to assess the injunctive norms and perceived descriptive norms for parenthood (see Tables 4, 5, respectively). Interaction effects between main effects and condition types were also examined. Only significant three and four-way interactions were included in the models. In addition, all lower-level interactions were also included for significant three-way interactions, and there were no significant four-way interactions. Covariates, such as marriage and parenthood status of the participants, were not tested for interaction effects. *Post hoc* differences were evaluated using the Bonferroni correction, and only comparisons involving differences in type of married couple were examined to reduce the experiment-wise error rate and to be consistent with the hypotheses of this study.

**TABLE 4:** Fixed effects for injunctive norms.

Predictor	df	*F*	*p*-value	Partial η^2^
Race	(1, 1001.61)	1.41	0.235	< 0.01
Education	(1, 1002.48)	1.43	0.233	< 0.01
Parenthood status	(1, 1001.63)	2.62	0.106	< 0.01
Marriage status	(1, 1002.10)	0.03	0.856	< 0.01
Age	(3, 1032.52)	10.58*	< 0.001	0.03
Gender	(1, 1032.90)	5.85*	0.016	0.01
Couple condition (CC)	(2, 1006.17)	95.20*	< 0.001	0.16
Participant sexual orientation (PSO)	(1, 1039.28)	116.28*	< 0.001	0.10
CC × Age	(6, 1006.17)	1.45	0.192	< 0.01
CC × Gender	(2, 1006.19)	3.49*	0.031	0.01
CC × PSO	(2, 1006.17)	74.57*	< 0.001	0.13
PSO × Gender	(1, 1001.11)	4.05*	0.045	< 0.01
PSO × Age	(3, 1007.93)	1.54	0.201	< 0.01
CC × PSO × Gender	(2, 1006.19)	19.13*	< 0.001	0.04
CC × PSO × Age	(6, 1006.17)	2.81*	0.010	0.02

Couple condition was the type of couple presented within the repeated measure items, i.e., heterosexual couples, lesbian couples, or gay couples. F statistics with p-values less than 0.05 were flagged using an asterisk (*).

**TABLE 5:** Fixed effects for perceived descriptive norms.

Predictor	df	*F*	*p*-value	Partial η^2^
Race	(1, 1008)	2.73	0.099	< 0.01
Education	(1, 1008)	0.25	0.617	< 0.01
Parenthood Status	(1, 1008)	< 0.01	0.989	< 0.01
Marriage Status	(1, 1008)	3.09	0.079	< 0.01
Age	(3, 1018.08)	5.58*	0.001	0.02
Gender	(1, 1018.27)	15.32*	< 0.001	0.01
Couple condition (CC)	(2, 1012)	866.67*	< 0.001	0.63
Participant sexual orientation (PSO)	(1, 1020.27)	4.46*	0.035	< 0.01
CC × Age	(6, 1012)	5.23*	< 0.001	0.03
CC × Gender	(2, 1012)	0.10	0.907	< 0.01
CC × PSO	(2, 1012)	4.72*	0.009	0.01

Couple condition was the type of couple presented within the repeated measure items, i.e., heterosexual couples, lesbian couples, or gay couples. F statistics with p-values less than 0.05 were flagged using an asterisk (*).

Two indicators were used to interpret the normative attitudes for parenthood. The first indicator was based on using the heterosexual condition as the reference group. Participants rating the norms for married lesbian and gay couples to pursue parenthood similarly to norms for heterosexual married couples would be viewed as indicative of lesbian and gay parenthood being normative. Secondly, the injunctive and descriptive norm outcomes were used as indicators by comparing the norms reported for lesbian and gay parenthood to a neutral point on these measures. For injunctive norm scales, this reference point was 3, which represented a neutral value. This point was selected because injunctive norms are based on the degree to which people think others should or should not perform an action (Cialdini et al., 1991; Rimal and Real, 2003), and this point is operationalized as the point where people would not agree or disagree that people should pursue parenthood. Thus, results significantly above 3 were considered as evidence for parenthood norms, and results significantly below 3 were considered as evidence of norms against parenthood. For descriptive norm scales, 50%, i.e., a score of 5, was utilized as a reference point. This point was selected because descriptive norms are the degree to which people think that others perform a behavior (Cialdini et al., 1991; Rimal and Real, 2003), and 50% was operationalized as the neutral reference point for this study because that was the point where those encouraged to pursue parenthood would not be a minority or a majority. Thus, percentages significantly greater than 50% were considered as evidence of stronger parenthood norms, and percentages less than 50% were considered as evidence of weak norms for parenthood.

## Results

The results are presented in three sections. First, the results for the preliminary analyses are given, next the results for injunctive norms for parenthood are presented, and finally, perceived descriptive norms for parenthood results are described.

### Preliminary Analyses

There were differences in demographic variables as a function of gender and sexual orientation (see Table 3). Significant differences in racial/ethnicity proportions were found for gender and sexual orientation, χ^2^(2) = 13.72, *p* = 0.001, φ = 0.12, χ^2^(2) = 7.62, *p* = 0.022, φ = 0.09, respectively. A greater proportion of women (73%) in this sample were white compared to men (62%), *z* = 3.69, *p* < 0.001, *h* = 0.23. A greater proportion of lesbian/gay adults (10%) in this sample were multiracial than were heterosexual individuals (6%), *z* = 2.77, *p* = 0.006, *h* = 0.17. There were no differences in the proportion of people in each age grouping as a function of gender or sexual orientation due to the method of sampling.

Differences were also found in the amount of education as a function of sexual orientation, *F*(1,1016) = 27.27, *p* < 0.001, partial η^2^ = 0.03 (see Table 3). Lesbian/Gay adults reported more education than did heterosexual adults, *p* < 0.001. No significant effects were found for gender or the interaction of gender or sexual orientation in education level.

There was a significant interaction effect between gender and sexual orientation for parenthood status, Wald χ^2^(1) = 4.91, *p* = 0.027. Heterosexual women (57%) were more likely to be parents than were heterosexual men (40%), *p* < 0.001, *h* = 0.34, and lesbian women (40%) were more likely to be parents than were gay men (15%), *p* < 0.001, *h* = 0.57. Based on Cohen’s *h*, this difference was larger for lesbian/gay individuals than heterosexual individuals.

The analyses also found differences as a function of sexual orientation for marriage status, Wald χ^2^(1) = 26.92, *p* < 0.001. Heterosexual participants (39%) were more likely to be married than were lesbian/gay participants (23%), *p* < 0.001, *h* = 0.35. No differences in marital status were found as a function of gender or the interaction between gender and sexual orientation. In the following results, race, education, parenthood status, and marital status were included as covariates, but none of the covariates were significantly associated with either injunctive or perceived descriptive norms.

### Injunctive Norms

#### Main Effects of Participant Sexual Orientation, Gender, and Age

There were also differences in how individuals reported injunctive norms, in general, as a function of participant sexual orientation, gender, and age, *F*(1,1039.28) = 116.28, *p* < 0.001, partial η^2^ = 0.10, *F* (1, 1032.90) = 5.85, *p* = 0.016, partial η^2^ = 0.01, *F*(3,1032.52) = 10.58, *p* < 0.001, partial η^2^ = 0.03, respectively. Lesbian/gay participants (*M* = 4.41, *SE* = 0.04) reported higher injunctive norms averaged across all types of married couples than did heterosexual individuals (*M* = 3.84, *SE* = 0.04), *p* < 0.001, *g* = 0.68. Women (*M* = 4.19, *SE* = 0.04) reported higher injunctive norms averaged across all types of married couples than did men (*M* = 4.07, *SE* = 0.04), *p* = 0.016, *g* = 0.15. Older adults (*M* = 3.89, *SE* = 0.05) reported lower injunctive norms averaged across all types of married couples than did early adults (*M* = 4.32, *SE* = 0.06), young adults (*M* = 4.18, *SE* = 0.05), and Younger Middle Adults (*M* = 4.13, *SE* = 0.05), *p* < 0.001, *g* = 0.51, *p* = 0.001, *g* = 0.35, *p* = 0.007, *g* = 0.29, respectively. There were no other significant differences as a function of age.

#### Main Effects of Married Couple Conditions

The LMM revealed that there was a significant difference in injunctive norms for married lesbian, gay, and heterosexual couple conditions, *F*(2,1006.17) = 95.20, *p* < 0.001, partial η^2^ = 0.16. Participants reported that married heterosexual couples (*M* = 4.46, *SE* = 0.02) ought to become parents more than married lesbian (*M* = 4.01, *SE* = 0.04) and gay (*M* = 3.91, *SE* = 0.04) couples, *p* < 0.001, *d* = 0.48, *p* < 0.001, *d* = 0.56, respectively. Participants also reported that married lesbian couples ought to become parents more than married gay couples, *p* < 0.001, *d* = 0.09.

#### Interaction Effects

The differences among injunctive norms were dependent upon the sexual orientation, gender, and age of the participants. Only results for higher-level interactions are described, for the sake of clarity, which should be interpreted as the main findings.

**FIGURE 1:**
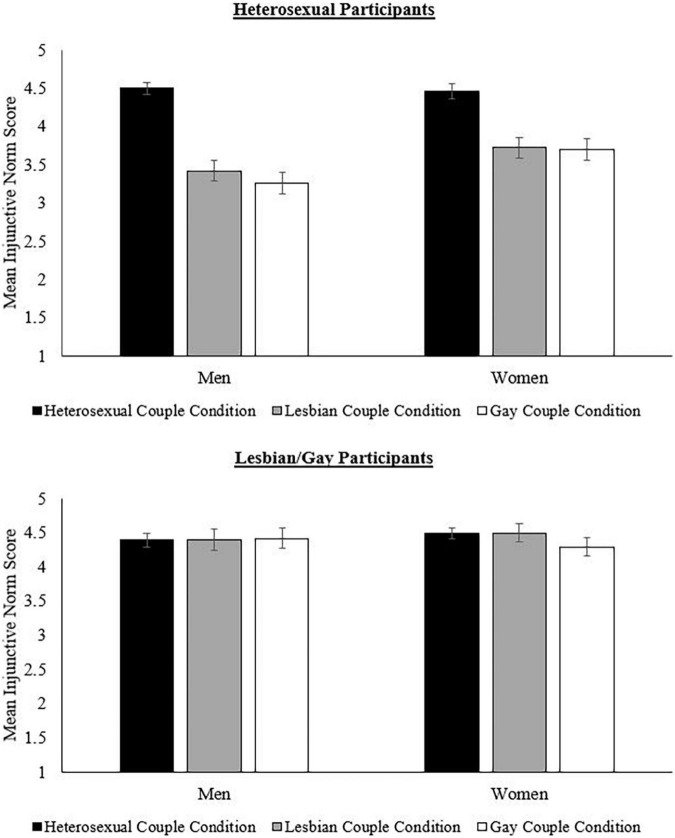
Injunctive norms as a function of gender, sexual orientation, and couple condition. Bars represent 95% CI.

The effects of sexual orientation on injunctive norms for parenthood of different types of married couples were dependent on the gender of the participant, *F*(2,1006.19) = 19.13, *p* < 0.001, partial η^2^ = 0.04 (Table 6 for statistics and Figure 1). Heterosexual women reported that heterosexual people should become parents more than gay and lesbian people, *p* < 0.001, *d* = 0.78, *p* < 0.001, *d* = 0.77, respectively, and heterosexual men also reported that heterosexual people should become parents more than gay and lesbian people, *p* < 0.001, *d* = 1.28, *p* < 0.001, *d* = 1.15, respectively. However, these differences were more pronounced for heterosexual men than for heterosexual women. Heterosexual men also reported that lesbian women should become parents more than gay men, *p* < 0.001, *d* = 0.14, but heterosexual women did not report any significant differences between lesbian and gay conditions, *p* > 0.999, *d* = 0.02.

**TABLE 6:** Differences in injunctive norms as a function of gender, sexual orientation, and couple condition.

Gender	Heterosexual couple condition		Lesbian couple condition	Gay couple condition	*F* (df)	*p*	Partial η^2^	Differences*^a^*	*p*	*d*
**Heterosexual participants**										
Men	M [95% CI] (SE)	4.50 [4.41, 4.59] (0.04)	3.42 [3.29, 3.56] (0.07)	3.26 [3.12, 3.40] (0.07)	125.78* (2,1006)	<0.001	0.20	HCC > LCC* HCC > GCC* LCC > GCC*	<0.001 <0.001 <0.001	1.15 1.28 0.14
Women	M [95% CI] (SE)	4.46 [4.37, 4.55] (0.05)	3.72 [3.59, 3.86] (0.07)	3.70 [3.55, 3.84] (0.07)	50.57* (2,1005.73)	<0.001	0.09	HCC > LCC* HCC > GCC* LCC ≈ GCC	<0.001 <0.001 >0.999	0.77 0.78 0.02
**Lesbian/gay participants**										
Men	M [95% CI] (SE)	4.39 [4.30, 4.49] (0.05)	4.40 [4.25, 4.55] (0.08)	4.42 [4.26, 4.57] (0.08)	0.10 (2,1004.74)	0.903	<0.01	HCC ≈ LCC HCC ≈ GCC LCC ≈ GCC	>0.999 >0.999 >0.999	0.01 0.02 0.01
Women	M [95% CI] (SE)	4.49 [4.40, 4.58] (0.04)	4.50 [4.37, 4.64] (0.07)	4.29 [4.14, 4.43] (0.07)	22.53* (2,1004.74)	<0.001	0.04	HCC ≈ LCC HCC > GCC* LCC > GCC*	>0.999 0.026 <0.001	0.01 0.21 0.19

^a^HCC represents the “Heterosexual Couple Condition,” LCC represents the “Lesbian Couple Condition,” and GCC represents the “Gay Couple Condition.” F statistics and pairwise comparison differences with p < 0.05 were flagged using an asterisk (*). Pairwise comparisons were corrected using the Bonferroni correction. Values for d represent Cohen’s d. For reference, injunctive norms were measured from 1 to 5 with numbers less than 3 representing that couples should not to become parents and values greater than three representing that couples ought to become parents.

Also, lesbian women reported that married heterosexual people should become parents more than married gay men, *p* = 0.026, *d* = 0.21, no differences between married heterosexual and lesbian conditions, *p* > 0.999, *d* = 0.01, and that married lesbian women ought to become parents more than married gay men, *p* < 0.001, *d* = 0.19. However, gay men reported no differences in injunctive norms for the pursuit of parenthood for married heterosexual individuals compared to married lesbian and gay individuals, *p* > 0.999, *d* = 0.01, *p* > 0.999, *d* = 0.02, respectively. Gay men also reported no difference in injunctive norms for the pursuit of parenthood between lesbian and gay conditions, *p* > 0.999, *d* = 0.01. Thus, differences among groups were greater for lesbian women than for gay men.

**FIGURE 2:**
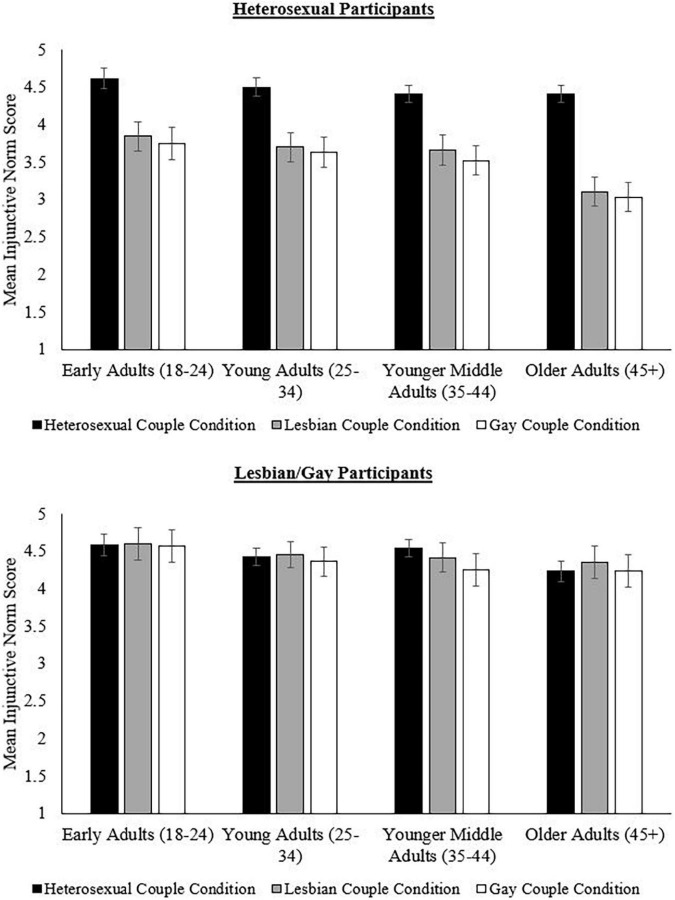
Injunctive norms as a function of age, sexual orientation, and couple condition. Bars represent 95% CIs.

In addition, the effects of the sexual orientation of participants on injunctive norms for parenthood of different types of married couples were also dependent on the age of the participant, *F*(6,1006.17) = 2.81, *p* = 0.010, partial η^2^ = 0.02 (see Table 7 and Figure 2). For heterosexual participants, the differences in injunctive norms between heterosexual and lesbian/gay conditions became more pronounced for those over the age of 44. In addition, younger middle adults (ages 35–44) were the only age group in which heterosexual participants responded that married lesbian women ought to become parents more than married gay men, *p* = 0.005, *d* = 0.13. However, lesbian/gay participants reported no differences in injunctive norms between any couple condition for all age categories, except younger middle adults (ages 35–44). For this age group, lesbian/gay participants reported that married heterosexual individuals ought to become parents more than married gay men, *p* = 0.033, *d* = 0.29. Younger middle adults were also the only age group in which lesbian/gay participants responded that married lesbian women ought to become parents more than married gay men, *p* = 0.002, *d* = 0.14. However, lesbian/gay participants in this age group reported no differences between married heterosexual and lesbian individuals, *p* = 0.663, *d* = 0.14.

**TABLE 7:** Differences in injunctive norms as a function of age, sexual orientation, and couple condition.

Age^1^	Heterosexual Couple Condition		Lesbian Couple Condition	Gay Couple Condition	F (df)	p	Partial η^2^	Differences	p	d
**Heterosexual participants**										
Early adults (18–24)	M [95% CI] (SE)	4.61 [4.48, 4.74] (0.07)	3.84 [3.64, 4.03] (0.10)	3.74 [3.53, 3.95] (0.11)	29.61* (2,1004.74)	<0.001	0.06	HCC > LCC* HCC > GCC* LCC ≈ GCC	<0.001 <0.001 0.121	0.81 0.88 0.08
Young adults (25–34)	M [95% CI] (SE)	4.50 [4.38, 4.62] (0.06)	3.70 [3.51, 3.89] (0.10)	3.63 [3.43, 3.82] (0.10)	32.94* (2,1004.74)	<0.001	0.06	HCC > LCC* HCC > GCC* LCC ≈ GCC	<0.001 <0.001 0.281	0.86 0.90 0.07
Younger middle adults (35–44)	M [95% CI] (SE)	4.41 [4.28, 4.53] (0.06)	3.66 [3.47, 3.86] (0.10)	3.52 [3.31, 3.72] (0.10)	32.11* (2,1004.74)	<0.001	0.06	HCC > LCC* HCC > GCC* LCC > GCC*	<0.001 <0.001 0.005	0.78 0.91 0.13
Older adults (45+)	M [95% CI] (SE)	4.41 [4.28, 4.53] (0.06)	3.10 [2.91, 3.29] (0.10)	3.03 [2.83, 3.23] (0.10)	82.75* (2,1009.27)	<0.001	0.14	HCC > LCC* HCC > GCC* LCC ≈ GCC	<0.001 <0.001 0.376	1.35 1.39 0.06
**Lesbian/gay participants**										
Early adults (18–24)	M [95% CI] (SE)	4.58 [4.45, 4.71] (0.07)	4.60 [4.39, 4.80] (0.11)	4.56 [4.34, 4.77] (0.11)	0.38 (2, 1004.74)	0.683	<0.01	HCC ≈ LCC HCC ≈ GCC LCC ≈ GCC	>0.999 >0.999 >0.999	0.02 0.02 0.03
Young adults (25–34)	M [95% CI] (SE)	4.42 [4.31, 4.54] (0.06)	4.45 [4.26, 4.63] (0.09)	4.36 [4.17, 4.55] (0.10)	2.02 (2,1004.74)	0.134	<0.01	HCC ≈ LCC HCC ≈ GCC LCC ≈ GCC	>0.999 >0.999 0.135	0.02 0.07 0.02
Younger middle adults (35–44)	M [95% CI] (SE)	4.54 [4.42, 4.67] (0.06)	4.41 [4.21, 4.61] (0.10)	4.25 [4.05, 4.46] (0.11)	6.79* (2,1004.74)	0.001	0.01	HCC ≈ LCC HCC > GCC* LCC > GCC*	0.663 0.033 0.002	0.14 0.29 0.14
Older adults (45+)	M [95% CI] (SE)	4.23 [4.09, 4.36] (0.07)	4.35 [4.14, 4.56] (0.11)	4.23 [4.01, 4.45] (0.11)	3.09* (2,1004.74)	0.046	0.01	HCC ≈ LCC HCC ≈ GCC LCC ≈ GCC	0.834 >0.999 0.063	0.13 0.01 0.10

^1^Ages included within operationalized age group are shown within parentheses. F statistics and pairwise comparison differences with p < 0.05 were flagged using an asterisk (*). Pairwise comparisons were corrected using the Bonferroni correction. For reference, injunctive norms were measured from 1 to 5 with numbers less than 3 representing that couples should not to become parents and values greater than three representing that couples ought to become parents.

#### Comparing Values to a Neutral Point

On the scale measuring injunctive norms, the neutral reference point was three. Any value above three represented that couples “ought to” become parents and any value below three represented that couples “should not” become parents. When comparing the mean values from the analyses to three (see Tables 5, 6 for means and 95% confidence intervals), it was clear most of the means were well above three. Thus, most respondents thought married lesbian and gay people ought to become parents, despite differences as a function of personal characteristics. Only two mean values were not significantly above three. Heterosexual adults over the age of 44 reported mean values for married gay men (*M* = 3.03, 95% CI [2.83, 3.23]) and married lesbian women (*M* = 3.10, 95% CI [2.91, 3.29]) that had 95% confidence intervals containing three.

### Perceived Descriptive Norms

#### Main Effects of Participant Sexual Orientation, Gender, and Age

There were also differences in how individuals reported perceived descriptive norms, in general, as a function of participant sexual orientation, gender, and age, *F*(1,1020.27) = 4.46, *p* = 0.035, partial η^2^ < 0.01, *F*(1,1018.27) = 15.32, *p* < 0.001, partial η^2^ = 0.01, *F*(3,1018.08) = 5.58, *p* = 0.001, partial η^2^ = 0.02, respectively. Lesbian/gay participants (*M* = 5.02, *SE* = 0.07) reported stronger perceived descriptive norms averaged across all types of married couples than did heterosexual individuals (*M* = 4.81, *SE* = 0.07), *p* = 0.036, *g* = 0.13. Women (*M* = 5.11, *SE* = 0.07) reported higher perceived descriptive norms averaged across all types of married couples than did men (*M* = 4.72, *SE* = 0.07), *p* < 0.001, *g* = 0.25. Older adults (*M* = 4.61, *SE* = 0.10) reported weaker perceived descriptive norms averaged across all types of married couples than did Early Adults (*M* = 5.15, *SE* = 0.11) and Young Adults (*M* = 5.08, *SE* = 0.09), *p* = 0.002, *g* = 0.34, *p* = 0.004, *g* = 0.30, respectively. There were no other significant differences as a function of age. In all, lesbian and gay adults, women, and younger adults reported stronger descriptive norms (averaged across all types of married couples) than did heterosexual adults, men, and older adults, respectively.

#### Main Effects of Married Couple Conditions

The LMM revealed that there was a significant difference in perceived descriptive norms for married lesbian, gay, and heterosexual couples, *F*(2,1012) = 866.67, *p* < 0.001, partial η^2^ = 0.63. Participants reported that they thought more married heterosexual couples (*M* = 6.96, *SE* = 0.06) would become parents than would married lesbian (*M* = 4.19, *SE* = 0.07) and gay (*M* = 3.60, *SE* = 0.06) couples, *p* < 0.001, *d* = 1.37, *p* < 0.001, *d* = 1.71, respectively. Participants also reported that more married lesbian couples would become parents than married gay couples, *p* < 0.001, *d* = 0.28.

#### Interactions Effects

**FIGURE 3:**
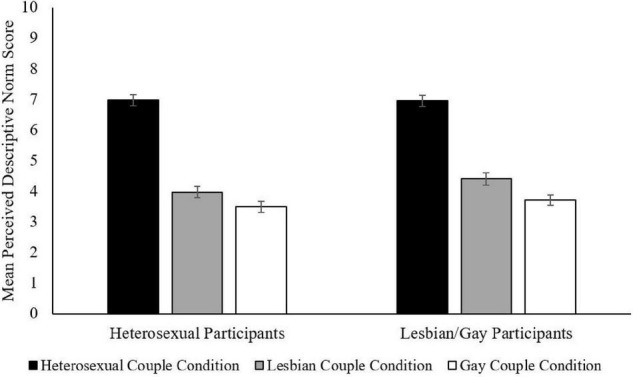
Perceived descriptive norms as a function of sexual orientation and couple condition. Bars represent 95% CIs.

**FIGURE 4:**
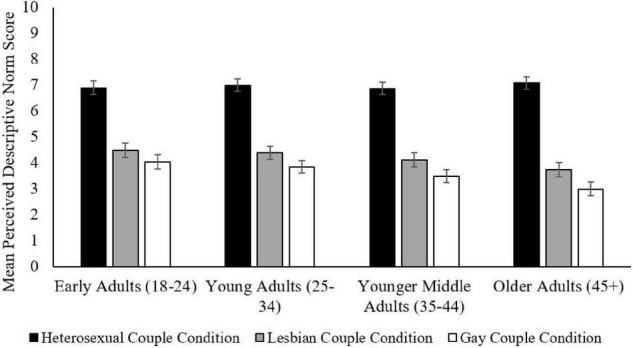
Perceived descriptive norms as a function of age and couple condition. Bars represent 95% CI.

The differences among perceived descriptive norms were dependent upon the sexual orientation and age of the participants *F*(2,1012) = 4.72, *p* = 0.009, partial η^2^ = 0.01, *F* (6,1012) = 5.23, *p* < 0.001, partial η^2^ = 0.03, respectively (see Tables 8, 9 for statistics and Figures 3, 4). When examining perceived descriptive norms for different couples to become parents by sexual orientation, both heterosexual and lesbian/gay participants reported that more married heterosexual people would become parents than married lesbian and gay people and that more married lesbian couples would become parents than married gay couples, *p* < 0.001 for all differences. However, lesbian/gay participants reported a less extreme difference in how many married heterosexual individuals would have children compared to married lesbian, *d* = 1.24, and gay individuals, *d* = 1.62, especially married lesbian women, than did heterosexual participants, *d* = 1.46 and *d* = 1.75, respectively (see Table 8 and Figure 3). However, lesbian/gay participants reported a more extreme difference between married lesbian and gay people, *d* = 0.32, than did heterosexual participants, *d* = 0.23.

**TABLE 8:** Differences in perceived descriptive norms as a function of sexual orientation and couple condition.

Participant sexual orientation		Heterosexual couple condition	Lesbian couple condition	Gay couple condition	*F* (df)	*p*	Partial η^2^	Differences	*p*	d
Heterosexual	M [95% CI] (SE)	6.97 [6.80, 7.13] (0.09)	3.97 [3.79, 4.16] (0.09)	3.49 [3.31, 3.66] (0.09)	479.88 (2, 1012)	<0.001	0.49	HCC > LCC* HCC > GCC* LCC > GCC*	<0.001 <0.001 <0.001	1.46 1.75 0.23
Lesbian/Gay	M [95% CI] (SE)	6.95 [6.78, 7.12] (0.09)	4.40 [4.21, 4.59] (0.10)	3.71 [3.53, 3.89] (0.09)	394.25 (2,1012)	<0.001	0.44	HCC > LCC* HCC > GCC* LCC > GCC*	<0.001 <0.001 <0.001	1.24 1.62 0.32

F statistics and pairwise comparison differences with p < 0.05 were flagged using an asterisk (*). Pairwise comparisons were corrected using the Bonferroni correction. For reference, perceived descriptive norms were measured from 0 to 10 with 10% intervals such that 0 = “0%,” 5 = “50%,” 10 = “100%.”

**TABLE 9:** Differences in perceived descriptive norms as a function of age and couple condition.

Age^1^		Heterosexual couple condition	Lesbian couple condition	Gay couple condition	*F* (df)	*p*	Partial η^2^	Differences	*p*	*d*
Early adults (18–24)	M [95% CI] (SE)	6.90 [6.64, 7.15] (0.13)	4.50 [4.22, 4.77] (0.14)	4.05 [3.79, 4.32] (0.14)	146.54* (2,1012)	<0.001	0.22	HCC > LCC* HCC > GCC* LCC > GCC*	<0.001 <0.001 <0.001	1.14 1.39 0.21
Young adults (25–34)	M [95% CI] (SE)	7.00 [6.77, 7.22] (0.12)	4.39 [4.13, 4.64] (0.13)	3.85 [3.61, 4.09] (0.12)	209.52* (2,1012)	<0.001	0.29	HCC > LCC* HCC > GCC* LCC > GCC*	<0.001 <0.001 <0.001	1.28 1.59 0.25
Younger middle adults (35–44)	M [95% CI] (SE)	6.87 [6.63, 7.11] (0.12)	4.12 [3.86, 4.39] (0.14)	3.49 [3.24, 3.74] (0.13)	221.05* (2,1012)	<0.001	0.30	HCC > LCC* HCC > GCC* LCC > GCC*	<0.001 <0.001 <0.001	1.34 1.70 0.29
Older adults (45+)	M [95% CI] (SE)	7.08 [6.83, 7.32] (0.12)	3.74 [3.47, 4.01] (0.14)	3.00 [2.45, 3.26] (0.13)	305.36* (2,1012)	<0.001	0.38	HCC > LCC* HCC > GCC* LCC > GCC*	<0.001 <0.001 <0.001	1.63 2.05 0.35

^1^Ages included within operationalized age group are shown within parentheses. F statistics and pairwise comparison differences with p < 0.05 were flagged using an asterisk (*). Pairwise comparisons were corrected using the Bonferroni correction. For reference, perceived descriptive norms were measured from 0 to 10 with 10% intervals such that 0 = “0%,” 5 = “50%,” 10 = “100%.”

When considering differences among perceived descriptive norms by age, every age category reported that more married heterosexual people would become parents than those who were lesbian and gay and that more married lesbian women would become parents than married gay men, *p* < 0.001 for all differences. However, differences in perceptions of how many married lesbian, gay, and heterosexual people become parents were more extreme among older participants (see Table 9 and Figure 4). This included differences between heterosexual and lesbian and gay conditions, as well as differences between lesbian and gay conditions.

#### Comparing Values to a Neutral Point

On the scale measuring descriptive norms, the neutral reference point was five. Any value above five represented that a majority of couples become parents and any value below five represented that only a minority of couples become parents. When comparing the mean values from the analyses to five (see Tables 8, 9 for means and 95% confidence intervals), it was apparent that participants, regardless of sexual orientation, gender, or age, responded that most heterosexual married couples would become parents together, while only a minority of married lesbian and gay couples would do so.

## Discussion

Parenthood is a common experience during human development (Jamison et al., 1979; Mueller and Yoder, 1997, 1999; Kopper and Smith, 2001; DeJean et al., 2012; Ashburn-Nardo, 2017). However, most prior research on normative aspects of parenthood has been done with heterosexual people. Now that lesbian and gay individuals can pursue marriages in same-sex relationships, it is important to examine the norms for parenthood among married same-sex couples. This study examined injunctive and perceived descriptive norms for parenthood as a function of gender and sexual orientation over multiple age groups, and sought to answer three questions: (1) How do norms for parenthood differ depending on the type of couple pursuing parenthood? (2) How do these differences depend on the sexual orientation, gender, and age of those responding to the norm measures? (3) What do these results indicate about the norms to pursue parenthood among married same-sex couples?

When considering the first question, the findings of this study completely coincided with expectations. Results showed that, for both injunctive and perceived descriptive norms, participants described norms for married different-sex couples to pursue parenthood that were stronger than norms for married same-sex couples. Also as expected, both types of norms for parenthood were stronger for married lesbian couples than for married gay couples. This implies that adults tend to endorse parenthood among different-sex couples to a greater extent than among same-sex couples and parenthood among lesbian couples more than among gay male couples.

Many of the hypotheses for the second question were also supported by these findings for both injunctive and perceived descriptive norms. Lesbian and gay individuals endorsed stronger norms for lesbian/gay parenthood than did heterosexual individuals, women endorsed stronger norms for lesbian and gay parenthood than did men, and younger participants endorsed stronger norms for lesbian and gay parenthood than did older participants. In all, this meant that lesbian and gay adults, women, and younger adults were more in favor of parenthood among married same-sex couples than were heterosexual adults, men, and older adults, respectively.

However, there were some unexpected findings in how participant age, gender, and sexual orientation were associated with differences in norms. One of the most unanticipated findings was that gay men reported no differences in how much they thought couples ought to become parents, regardless of the couple type. However, even though both lesbian and gay adults endorsed surprisingly strong norms for all types of married couples, lesbian women viewed parenthood for married gay couples less favorably than parenthood among married different-sex and lesbian couples. This finding supports the idea that lesbian women ascribe gender norms to parenthood (e.g., “women should be involved in childcare more than men” or that “women ‘should be’ mothers”) (Mueller and Yoder, 1997, 1999; DeJean et al., 2012; Ashburn-Nardo, 2017). In essence, gender and attitudes about parenthood are explicitly tied together, and thus, combining lesbian and gay individuals into a unified group for the purposes of understanding prospective parenthood may not be the best practice (Gato et al., 2017; Tate et al., 2019).

In addition, participants between the ages of 35–44 acted differently than expected. Lesbian and gay individuals in this age group endorsed more extreme differences between married gay couples and married different-sex and lesbian couples compared to both younger and older lesbian and gay participants. Heterosexual participants in this age group also reported more differences between married lesbian and gay couples in how much they ought to become parents than did other age groups of heterosexual adults. This further supports the idea that the age group from 35 to 44 years old is a distinct group in Middle Adulthood (Costa and Bidell, 2016).

Finally, for the third research question, it was expected that participants would endorse parenthood as something that some married same-sex couples should do, but also something that not that many lesbian and gay people would do. The findings of this study supported this hypothesis. Despite differences as a function of sexual orientation, gender, and age, most people reported that parenthood was an aspect of life that married same-sex couples ought to pursue. Only older heterosexual participants responded differently. Moreover, these results showed that parenthood was also something that most participants believed that only a minority of married same-sex couples, especially male couples, would achieve during their marriage, regardless of participants’ sexual orientation, gender, and age. This is also reflective of the parenthood desire and expectation gap found in the literature, parenthood is an aspect of life that many gay and lesbian individuals want, but do not believe that they can actually achieve (Shenkman, 2012; Tate and Patterson, 2019).

Based on these results, it was clear that injunctive normative beliefs did not underlie the view that only a minority of married same-sex couples will become parents together. Overwhelmingly, individuals responded positively to the idea of lesbian and gay parenthood. Thus, it may be possible that perceptions of how many married same-sex couples will actually become parents (i.e., descriptive norms) may be derived from other aspects of people’s lives, such as a lack of exposure to parents raising children in same-sex couples (Costa et al., 2015), an acknowledgment of the difficulties experienced by same-sex couples in the pursuit of parenthood (Allen and Demo, 1995; Brown et al., 2009; Rabun and Oswald, 2009; Riskind et al., 2013; Blake et al., 2017), and/or an understanding that achieving parenthood is more accessible for lesbian women than for gay men (Blake et al., 2017; Gato et al., 2017).

Overall, the results of this study were generally consistent with expectations. Norms for the pursuit of parenthood were stronger for married different-sex couples than for married same-sex couples, and these norms were also stronger for married lesbian couples than married gay couples. Differences as a function of couple sexual orientation were much more pronounced for participants who were older, heterosexual, and male. Finally, most participants reported that married same-sex couples ought to pursue parenthood, but that only a minority of these couples would actually become parents together. These results have implications for understanding prospective parenthood among lesbian and gay populations.

### Contributions and Implications

This study sheds new light on the perspectives of lesbian and gay adults on lesbian and gay parenthood. Before this study, little to no research had asked lesbian and gay adults about their thoughts about the pursuit of parenthood by other lesbian and gay people. Most of the research has focused on how lesbian and gay adults think about their own parenthood (Gato et al., 2017). This literature has found that lesbian and gay adults are far less likely than heterosexual adults to desire, expect, or intend to pursue parenthood (Gato et al., 2017; Tate et al., 2019; Tate and Patterson, 2019). Results of this study showed that most lesbian and gay adults thought that married same-sex couples ought to become parents. More work needs to be done to explain the apparent discrepancy between lesbian and gay adults thinking that other lesbian and gay people should become parents, but also thinking that they themselves should not be parents. Maybe lesbian and gay people think “if someone is married, they should become a parent,” but do not believe that they themselves will ever get married (Tate and Patterson, 2019)? Based on findings within this study, this discrepancy may derive from the fact that heterosexual adults are more likely to be married than lesbian and gay adults.

This study also has implications for the mechanisms of disparities in parenthood aspirations as a function of sexual orientation. Many studies have found that lesbian and gay adults have lower aspirations for parenthood in comparison to heterosexual adults (Riskind and Tornello, 2017; Tate et al., 2019; Tate and Patterson, 2019). Many reasons have been put forth as possible sources of this disparity such as issues of discrimination, accessibility, and a lack of social support (Berkowitz and Marsiglio, 2007; DeMino et al., 2007; Gianino, 2008; Goldberg, 2010; Goldberg et al., 2012). However, recent work has shown that lesbian and gay adults are also less likely to expect to get married in the United States compared to heterosexual adults (Tate and Patterson, 2019). The current study found that lesbian and gay individuals reported that married people who are like them should become parents. If a norm for parenthood exists within a married context, then lesbian and gay adults who get married or plan to be married would theoretically experience normative pressures to pursue parenthood (DeJean et al., 2012; Ashburn-Nardo, 2017). Thus, using this normative theoretical framework, disparities in expectations for marriage may underlie disparities in parenthood aspirations.

This work introduces a social normative framework for investigating lesbian and gay parenthood. A social normative framework for parenthood has not been heavily utilized in the study of lesbian and gay populations, especially for those who are lesbian (Kranz et al., 2018). The current study found that lesbian and gay adults may be assimilating to the existing normative structure surrounding parenthood. When examining minority populations, assimilation is often regarded as a way of gaining power, status, and belonging within a culture designed by the majority to sustain these “normative” systems (Bowleg, 2012). In this case, it could be that lesbian and gay adults support these norms for married couples as a way of navigating these systems to gain power, status, and/or belonging. However, lesbian and gay populations tend to expand on the norms for parenthood that they are also upholding (Rabun and Oswald, 2009; Sumontha et al., 2017), a process this paper puts forth as “augmented assimilation.” Current results show that lesbian and gay adults are assimilating to norms of parenthood and also augmenting the concept by incorporating new people and new behavioral patterns into it. More work should be done to examine other ways in which lesbian and gay adults both ascribe to norms, but also expand upon them.

Overall, this study has made significant contributions to the literature on prospective parenthood among lesbian and gay populations. Findings from this study provided new information on the viewpoints that lesbian and gay adults hold toward prospective parenthood for other lesbian and gay people. This work also provided further information about the mechanisms of parenthood aspiration disparities as a function of sexual orientation. Finally, this research produced more evidence that lesbian and gay adults are assimilating to norms for parenthood that have historically only been applicable to heterosexual populations.

### Strengths and Limitations

This study had several strengths. The sample size was substantial, especially considering its use of repeated measures. The results also replicated work on norms for heterosexual parenthood in a novel way and provided new findings on the norms for lesbian and gay parenthood (Mueller and Yoder, 1997, 1999; DeJean et al., 2012; Ashburn-Nardo, 2017). The sample was also quite diverse in terms of age, race, education, and location, with participants from all states, except Alaska, represented in the data.

In addition, the use of IRT analyses to evaluate the items assessing injunctive norms provided strong evidence of their reliability. The analyses found that the items fit together extremely well, resulting in overfit statistics (Embretson and Reise, 2000). Thus, IRT findings justified both the decision to average the four items together for this study and the use of the items to measure injunctive norms in general. Future work should examine whether these items are reliable over time and across a variety of samples.

Moreover, this study laid the foundation for future work. Because these issues have not been heavily researched for lesbian and gay populations (Kranz et al., 2018), the findings provided insights into the norms for the pursuit of parenthood in the United States as a function of sexual orientation, gender, and age. Based on the current study, new work can examine the nuances within the United States and norms within other countries using the United States as a comparison. In this way, the results can provide a useful basis for future research.

Despite these strengths, this research also had limitations. The study did not include data from plurisexual and transgender individuals. Therefore, their experiences and voices were not heard. In addition, the sample cannot be regarded as representative of any population. However, the research design allowed for oversampling of older lesbian and gay adults. Moreover, there was no measure of implicit bias that individuals may have about lesbian and gay people. Thus, it was not possible to evaluate the possible role of implicit bias in these results. Moreover, data were collected before the COVID-19 pandemic, which may have impacted norms for everyone.

In addition, the method utilized to assess norms was based on the use of written materials. In other words, participants were asked how they felt about “hypothetical” couples with a “hypothetical” description. However, this method may have weaknesses (Aguinis and Bradley, 2014). For instance, utilizing pictures or videos and conjoint analysis, where all conditions are presented at the same time and participants make active comparisons, may have resulted in different findings because the couples go from being “paper people” to being more concrete and “real” (Aguinis and Bradley, 2014). Future work should examine the generality of these findings utilizing other methods.

Overall, this study was able to highlight, in general, how age, gender, and sexual orientation are associated with norms about the pursuit of parenthood by married heterosexual, lesbian, and gay couples. However, it should be noted that there are additional possible confounds related to norms that should be examined in the future, such as characteristics related to interdependence including religiosity, conservatism, and rurality (Markus and Conner, 2014). Moreover, the analyses did not fully examine marriage and parenthood status of the participants, which may have more nuanced findings. Nevertheless, this study has laid the foundation to examine those potential confounds through the lenses of age, gender, and sexual orientation in future work.

## Conclusion

Parenthood decisions are some of the most consequential choices that people make (Hoffman, 1975; Langdridge et al., 2005; Jaffe and Diamond, 2011). Normative social influence plays a large role in shaping the parenthood decisions that heterosexual people make (Jamison et al., 1979; Mueller and Yoder, 1997, 1999; Kopper and Smith, 2001; DeJean et al., 2012; Ashburn-Nardo, 2017). It has also been found that many lesbian and gay individuals choose not to become parents, and that they even aspire to parenthood less than their heterosexual counterparts (Riskind and Tornello, 2017; Tate et al., 2019). However, norms for parenthood among same-sex couples have not been the focus of most research.

Results revealed that adults believe that married different-sex couples both ought to and will become parents more than married same-sex couples, and that married lesbian couples both ought to and will become parents more than married gay couples. However, these findings were dependent to some extent on the sexual orientation, gender, and age of the who were answering these questions. Most people thought that parenthood was something that married same-sex couples ought to do, despite differences as a function of sexual orientation, gender, and age, but that only a minority of these couples would actually do it. This was indicative of overwhelming positive injunctive norms for lesbian and gay parenthood, but a relatively low perception of how many married same-sex couples would pursue parenthood together.

In all, this research provided a glimpse into how Americans are envisioning family formation among same-sex couples today and gave some reasons to believe that norms for lesbian and gay parenthood are present, especially among young Americans. Adults tended to respond positively to lesbian and gay parenthood, which may be an indication that bias against lesbian and gay people is not the best explanation for the relatively low perception of how many married same-sex couples would eventually become parents. This study contributed to the literature on lesbian and gay prospective parenthood and provided a foundation for future research. Overall, at least in the United States, the normative context for family formation in married same-sex couples seems to be overwhelmingly positive.

## Data Availability Statement

The raw data supporting the conclusions of this article will be made available by the authors, without undue reservation.

## Ethics Statement

The studies involving human participants were reviewed and approved by the University of Virginia Social Sciences Institutional Review Board. The patients/participants provided their written informed consent to participate in this study.

## Author Contributions

DT contributed to conception and design of the study, collected the data, performed the statistical analysis, wrote all sections of the manuscript, contributed to manuscript revision, read, and approved the submitted version.

## Conflict of Interest

The author declares that the research was conducted in the absence of any commercial or financial relationships that could be construed as a potential conflict of interest.

## Publisher’s Note

All claims expressed in this article are solely those of the authors and do not necessarily represent those of their affiliated organizations, or those of the publisher, the editors and the reviewers. Any product that may be evaluated in this article, or claim that may be made by its manufacturer, is not guaranteed or endorsed by the publisher.
